# Effects of Pyrolysis on High-Capacity Si-Based Anode of Lithium Ion Battery with High Coulombic Efficiency and Long Cycling Life

**DOI:** 10.3390/nano12030469

**Published:** 2022-01-29

**Authors:** Yonhua Tzeng, Cheng-Ying Jhan, Yi-Hsuan Wu

**Affiliations:** Institute of Microelectronics, Department of Electrical Engineering, National Cheng Kung University, One University Road, Tainan 70101, Taiwan; m10506126@gmail.com (C.-Y.J.); d980372@gmail.com (Y.-H.W.)

**Keywords:** silicon, pyrolysis, Lithium ion battery, anode, graphitic carbon, high initial coulomb efficiency

## Abstract

We report a facile pyrolysis process for the fabrication of a porous silicon-based anode for lithium-ion battery. Silicon flakes of 100 nm × 800 nm × 800 nm were mixed with equal weight of sodium carboxymethyl cellulose (CMC) and styrene butadiene rubber (SBR) as the binder and the conductivity enhancement additive, Ketjen Black (KB), at the weight ratio of silicon–binder–KB being 70%:20%:10%, respectively. Pyrolysis was carried out at 700 °C in an inert argon environment for one hour. The process converts the binder to graphitic carbon coatings on silicon and a porous carbon structure. The process led to initial coulombic efficiency (ICE) being improved from 67% before pyrolysis to 75% after pyrolysis with the retention of 2.1 mAh/cm^2^ areal capacity after 100 discharge–charge cycles at 1 A/g rate. The improved ICE and cycling performance are attributed to graphitic coatings, which protect silicon from irreversible reactions with the electrolyte to form compounds such as lithium–silicon–fluoride (Li_2_SiF_6_) and the physical integrity and buffer space provided by the porous carbon structure. By eliminating the adverse effects of KB, the anode made with silicon-to-binder weight ratio of 70%:30% exhibited further improvement of the ICE to approximately 90%. This demonstrated that pyrolysis is a facile and promising one-step process for the fabrication of silicon-based anode with high ICE and long cycling life. This is especially true when the amount and choice of conductivity enhancement additive are optimized.

## 1. Introduction

Silicon is abundant and used in large quantity in the global semiconductor industry. It exhibits a very high theoretical capacity of 4200 mAhg^−1^ as an anode for lithium-ion battery with a very low oxidation-reduction potential of less than 0.5 V vs. Li/Li^+^, and low reactivity with electrolytes [1]. However, silicon also exhibits high electrical resistivity and suffers from a very large change (about 300%) in volume due to the formation of silicon-lithium compounds during lithiation. The volume changes often cause an anode to lose its physical integrity and charge–discharge capacity. The formation of SEI before silicon is primed to store charges by lithiation results in a significant irreversible loss of lithium [2]. An initial coulombic efficiency (ICE) of 80% causes the loss of 20% of the lithium that reacts to form SEI during the first-time lithiation of silicon. A CE of 99% during the subsequent cycles results in additional 1% loss of lithium that participates in the formation of lithium-silicon compound in each charge–discharge cycle. In order to preserve a limited quantity of lithium in a packaged battery for achieving a long cycling life, the coulombic efficiency is desired to be as close to 100% as possible. A packaged battery fails prematurely when the limited amount of lithium is consumed. [3] Among mechanisms causing the loss of lithium during charge–discharge cycling, cracked and pulmerized silicon in the anode exposes fresh silicon surfaces, which need to react with the electrolyte to form new SEI, also consumes lithium [4,5]. Under severe conditions, silicon cracks result in electrical insulation between broken silicon pieces and the subsequent loss of electron conduction paths and the silicon capacity. Stresses induced by the volume changes due to the formation of different phases of silicon–lithium compounds are the main cause of silicon cracks. Therefore, reserved room in an as-fabricated silicon-based anode is desirable for the accommodation of the initial increase in the volume of the anode when silicon–lithium compounds are formed for the first time.

Many attempts have been reported to overcome barriers against a long-life silicon-based anode for lithium-ion battery. Chemical passivation layers were coated on the surface of the silicon powder followed by an electrically conductive carbon layer [6,7,8,9]. The chemical passivation layers reduce undesirable reactions between silicon surface and the electrolyte, and thus enhance the coulombic efficiency and cycling life. [10] Core–shell structures were often applied, too. They rely on carbon materials wrapping around silicon particles to enhance the conductivity of the silicon powder, to reduce the agglomeration of silicon particles, to resist against volume expansion of active silicon material during the formation of silicon–lithium compounds, and to inhibit the contact and chemical reactions of silicon with the electrolyte. Owing to synergistic effects, this class of structures significantly contribute to the improvement of the performance of lithium-ion batteries. [11] The Stober method was applied to synthesize a uniform oxide layer on the silicon surface followed by coating a carbon layer on the outer surface. The silicon dioxide is then etched away to form a Si/void/C yolk-shell structure. It leaves a vacant space between the silicon and the carbon layer for accommodating the silicon volume expansion. The porous outer shell of amorphous silicon dioxide and carbon provide both mechanical strength and enhanced electrical conductivity. One example is the metal reduction of silicon aluminum alloy to form porous silicon with voids for silicon volume expansion. [12] In summary, it is helpful for the performance of silicon-based anode if one can fabricate an anode, which allows silicon volume expansion while maintaining physical integrity and electrical conductivity among silicon particles and between silicon and the current collector.

Small silicon particles with high surface-to-volume ratios are more effective in fending off stresses induced in silicon particles during formation of silicon–lithium compounds and their decomposition, which cause silicon volume changes [13,14]. Silicon powders of 100 nm size or smaller are more popular for the fabrication of silicon-based anode. The costs of manufacturing and purifying a large quantity of silicon nanoparticles are significant. Therefore, it is desirable to pursue the possibility of applying low-cost micron-size silicon powder for commercial silicon-based anode.

Silicon-based anode usually relies on some kind of binder that adheres to silicon particles for forming a solid electrode. PVDF is among widely used binders with strong electrochemical corrosion resistance. [15] However, *N*-methylpyrrolidone (NMP), which is often used as a solvent for PVDF, is harmful to the environment. At present, water-based binders like CMC and PAA [16] are widely used in silicon anodes. CMC film is brittle and sometimes can be toughened by SBR additive [17].

In this paper, silicon powder of the nominal dimensions of 100 nm × 800 mm × 800 nm that is recycled from manufacturing waste of silicon semiconductor industry is used with a mixed binder consisting of CMC and SBR in a ratio of 70%:30% by weight. The flat silicon flakes pile up irregularly and at different angles leaving much room between silicon flakes for the binder to fill and form a solid anode. The applied pressure during the formation of an anode film on a copper current collector promotes the mutual physical contacts between individual silicon flakes paving ways for electrical conduction among silicon flakes and with the current collector. The as-fabricated anode contains silicon, which exhibits insulating surface oxide. The binder is not conductive, neither. The anode is then pyrolyzed in inert environments at 700 °C for 1 h followed by slow cooling down to room temperature at a rate of 5 °C/min. An optimized pyrolysis process converts the binder that is in contact with silicon to a coating of electrically conductive graphitic carbon on silicon surface forming a conductive 3D silicon networks. Pyrolyzed binder exhibits voids, which are in the space among neighboring silicon flakes. Voids serve as reserved space for silicon volume expansion during the formation of silicon–lithium compounds and reduce the induced stress due to the volume changes of silicon flakes. The graphitic carbon coatings on silicon reduce reactions between silicon and the electrolyte leading to a higher initial coulombic efficiency of about 90%. The conductive 3D network of silicon and pyrolyzed binder of CMC and SBR provides stable and low resistance electron transport. A silicon-based anode without conductivity enhancement additive exhibits good cycling performance. By the addition of 5.7 wt% conductivity enhancement additives, the anode exhibits high capacity of 1000 mAh/g at a discharge–charge current of 2 A/g.

## 2. Materials and Methods

### 2.1. Chemicals

AUO Crystal Corporation in Taichung City, Taiwan, ROC, supplied silicon flakes of about 100 nm thick and 800 nm in length and width. It is a silicon wafer manufacturing company in Taiwan. The silicon flakes are part of silicon containing waste slurry generated by cutting silicon ingots and from other silicon wafer manufacturing processes. The company uses an economic and proprietary chemical process to recover and purify the silicon flakes from the slurry.

Battery-grade electrolyte, i.e., 1 M LiPF_6_ dissolved ethylene carbonate (EC), diethyl carbonate (DEC), and Dimethyl carbonate (DMC) in a 1:1:1 ratio by volume with 10 wt% Fluoroethylene carbonate (FEC) was purchased from Taiwan Hopax Chems. MFG. Co., Taipei, Taiwan. Ketjen Black (KB), model EC 600JD and Super P, model Super P were obtained from Eubiq Technology Co. (Taipei, Taiwan) as conductive agent.

### 2.2. Fabrication of Coin Half Cells with Si-Based Anode

Silicon flakes were mixed with sodium carboxymethyl cellulose (NaCMC) and Styrene-Butadiene (SBR) with a weight ratio of 70%:15%:15%. The slurry was stirred homogenously, and then applied on a 10-μm thick copper foil by a doctor blade. The thickness of the anode excluding the copper current collector was 15–20 µm.

After the electrode dried at 80 °C for 12 h, the electrode was cut into small pieces with a diameter of 12 mm. Then, the electrode was put into a two-inch quartz tubing. After an initial purge by argon (200 sccm), the reactor temperature was increased to 700 °C. It was held for 60 min in argon atmosphere, then the reactor was cooled down in argon at a rate of 5 °C/min.

For a comparison, anodes with silicon flake, binder (CMC/SBR), conductivity enhancement agent Ketjen Black (KB) by a weight ratio of 70%:20%:10% were also fabricated. These anodes were processed either with or without pyrolysis for comparison.

The electrodes were put into an Ar-filled glove box with residual oxygen and moisture contents of less than 0.5 ppm to assemble the coin cells. Lithium metal was used as the counter/reference electrode. The electrolyte was 1 M LiPF_6_ dissolved in ethylene carbonate (EC), diethyl carbonate (DEC), Dimethyl carbonate (DMC) by 1:1:1 volume ratio and with 10 wt% Fluoroethylene carbonate (FEC).

### 2.3. Materials Characterization

The morphology and structure of materials were observed by means of scanning electron microscopy (SEM, Hitachi-SU8000, Taipei, Taiwan) and scanning Transmission Electron Microscopy (JEOL JEM-2100F Cs STEM, Taipei, Taiwan) with an acceleration voltage of 200 kV. A Horiba Scientific (Taipei, Taiwan) Raman system with a green laser at 532 nm and laser power at 450 mW was used to measure Raman spectra. The laser beam was focused on the sample surface in an area of about 10 µm in size. Raman spectra reveal the nanostructures of the sample. XRD (Bruker AXS Gmbh, Arlsruhe, Germany) was used to analyze the phase of the pyrolytic silicon-based anode. The N_2_ adsorption/desorption isothermals of electrodes were performed at 77 K with the specific surface area calculated from the Brunauer–Emmett–Teller (BET) plot of the N_2_ adsorption isothermal.

### 2.4. Test Cells

The charge–discharge cycling was analyzed by a battery testing system (BAT-750B). The cells were cycled at a voltage window between 0.01 V and 1.50 V versus Li^+^/Li at 0.2 A/g for the first three cycles and 1.0 A/g for the following cycles. The specific capacity refers to the mAh per gram of silicon active material in the anode.

Cyclic voltammetry (CV) and electrochemical impedance spectroscopy (EIS) was measured using Autolab (Metrohm AUTOLAB BV, Taipei, Taiwan). The CV measurement used a scanning rate of 0.1 mV/s at the room temperature. The electrochemical impedance spectroscopy measurements were recorded at a frequency of 0.1–100 kHz.

## 3. Results and Discussion

**Figure 1:**
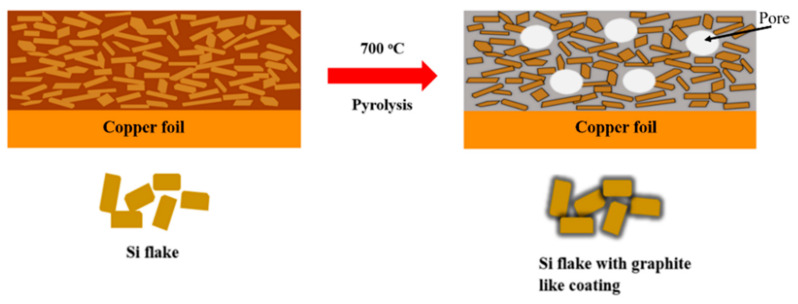
Schematic diagrams of silicon-based anode before and after pyrolysis in argon at 700 °C.

Figure 1 shows schematically an anode made of silicon flakes and binder on a copper foil before and after a pyrolysis of the anode in an inert argon environment at 700 °C. The pyrolysis converted the binder of a mixture of CMC and SBR to graphitic carbon coatings on individual silicon flakes which are embedded in a porous carbon structure. Pores are shown as white circles.

**Figure 2:**
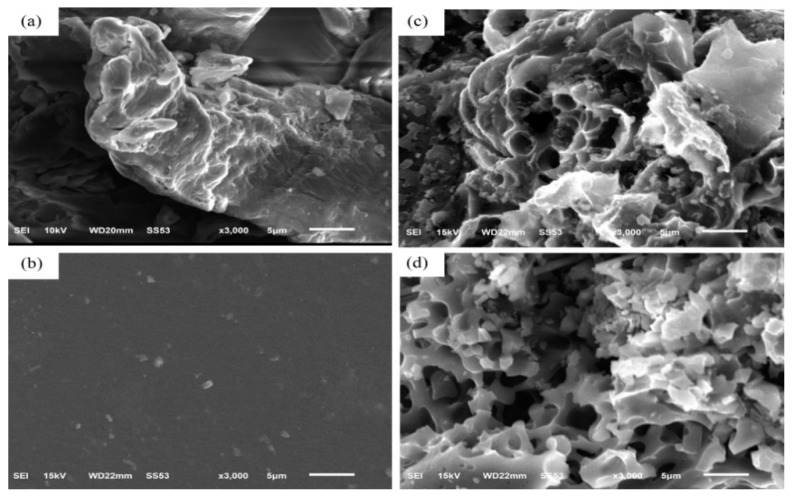
SEM images of (**a**) Carboxymethylcellulose (CMC) powder, (**b**) Styrene–Butadiene Rubber (SBR) film, (**c**) CMC—after pyrolysis, (**d**) SBR—after pyrolysis.

Figure 2a shows a SEM image of CMC after heating at 60 °C in vacuum to remove moisture. Figure 2b shows a SEM image of a SBR solution after having been placed in a vacuum oven at 80 °C for 8 h. These samples were subjected to pyrolysis in argon at 700 °C for 1 h; porous carbon materials are shown in Figure 2c,d, respectively. Pyrolysis process produced CO_2_, CO, C_2_H_4_O_2_, and CH_4_ gases [18]. The weight loss by the pyrolysis of these CMC and SBR binders were 85% and 87%, respectively.

**Figure 3:**
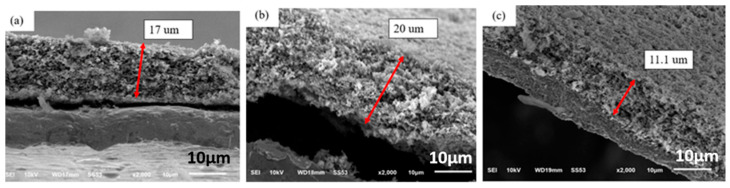
Cross-sectional SEM images of electrodes made of silicon flake and binder of the weight ratio of 7:3. (**a**) Before pyrolysis, (**b**) After pyrolysis, (**c**) After heat treatment at 600 °C in air.

Figure 3 shows cross-sectional SEM images of electrodes made of silicon flake and binder of the weight ratio of 70%:30%. Figure 3a shows an as-fabricated anode. Figure 3b shows the cross-sectional morphology of anodes after pyrolysis at 700 °C in argon for 1hr. The anode shown in Figure 3b was subjected to an oxidation process in air at 600 °C for 2 hr in order to remove amorphous carbon produced by pyrolysis in argon at 700 °C. Figure 3c shows the SEM cross-sectional image of the oxidized anode. Figure 3a shows that the thickness of the as-fabricated electrode is about 17 µm. After the pyrolysis process, the electrode lost about 25.8 wt%, but the thickness of the electrode did not decrease, but, instead increased from 17 µm to about 20 µm as shown in Figure 3b. Gaseous by-products generated by the pyrolysis of the binders created an internal pressure for the slight volume expansion and the formation of pores in the electrode.

The oxidation of the electrode at 600 °C in air atmosphere for 2 h removed amorphous carbon from the binder which had been subjected to pyrolysis in argon. The thickness of the electrode decreased significantly from 20 µ to 11.4 µm as shown in Figure 3c. The dark black surface of the anode changed to light coffee color that is similar to that of silicon flakes.

**Figure 4:**
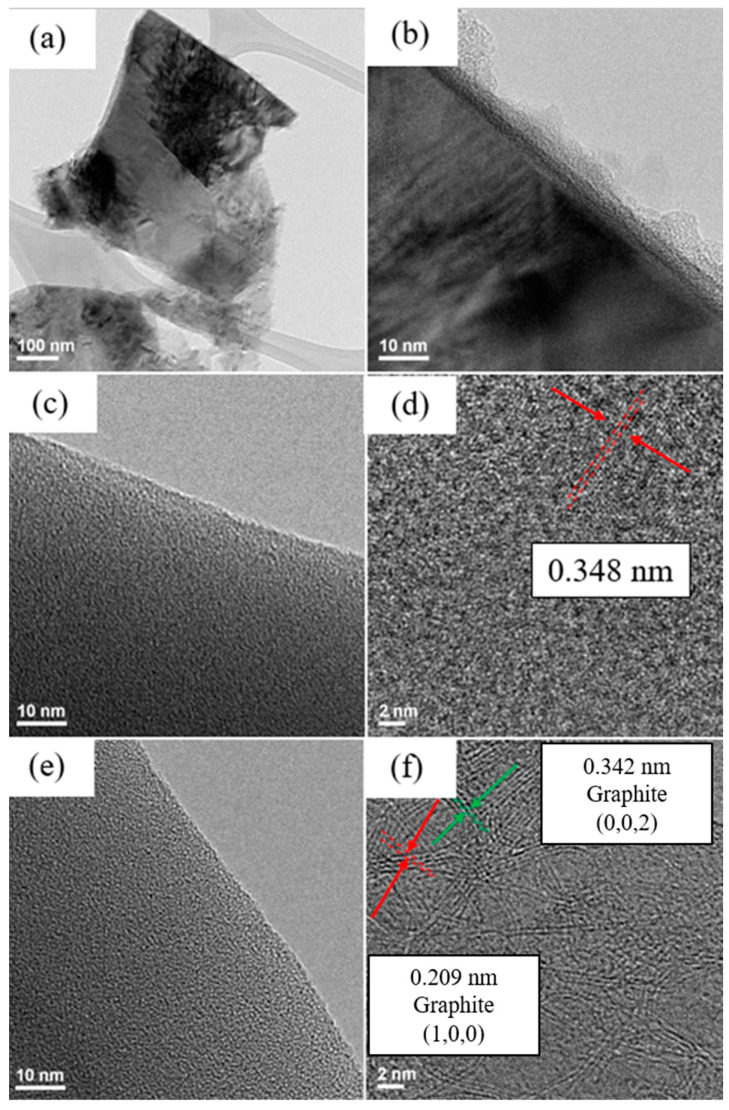
TEM images of (**a**,**b**) pristine silicon flakes, (**c**) pristine CMC, (**d**) pyrolytic CMC, (**e**) pristine SBR, and (**f**) pyrolytic SBR.

**Figure 5:**
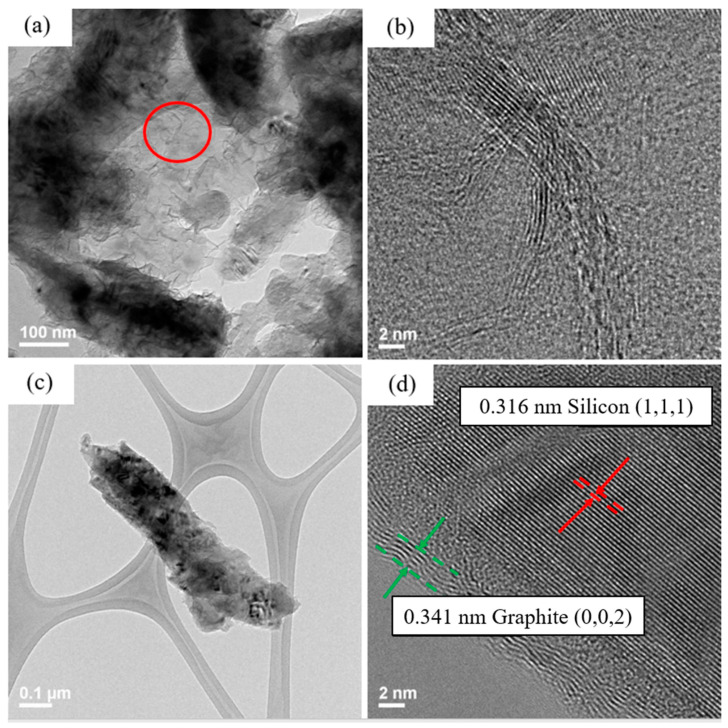
TEM images of (**a**) pyrolytic silicon anode, (**b**) pyrolytic binder, and (**c**,**d**) silicon flakes after pyrolysis.

Figure 4 and Figure 5 show TEM images of silicon flakes and binders before and after pyrolysis. Figure 4a,b shows silicon flakes. Figure 4b shows that a 3–8 nm oxide layer are present on the surface of silicon flake. Figure 4c,e show the morphology of CMC and SBR binders without pyrolysis, respectively. Both CMC and SBR exhibit amorphous nature. Figure 4d,f show that, after pyrolysis, CMC and SBR exhibited partial crystalline structural arrangements. The carbon matrix is considered to contribute to the overall conductivity of the electrode. The pyrolytic SBR shown in Figure 4f exhibits a higher degree of structural arrangement than that of CMC shown in Figure 4d. Long chain-like SBR molecules were converted by the pyrolysis process to a more crystalline carbon structure than that of CMC.

Pyrolytic electrode was dissolved in alcohol and subjected to ultrasonic vibration. The solution was then dropped on a copper net and dried in a vacuum oven. The microscopic structure of the silicon powder and the pyrolytic binders were analyzed by TEM images shown in Figure 5.

The darker portion of the TEM image shown in Figure 5a is silicon flake. The lighter part is amorphous and ordered carbon produced by pyrolysis. Shown in Figure 5b is a TEM image of the carbon structure after pyrolysis showing ordered carbon structure embedded in the disordered carbon. Orderly arranged carbon material is believed to contribute to the electrical conductivity of the anode. A silicon flake after pyrolysis is shown in Figure 5c with the surface layers shown in Figure 5d. Binder that adhered to the surface of silicon flake was converted to graphitic carbon coatings on silicon by the pyrolysis process. Graphitic carbon coating on silicon is desirable for reducing irreversible reactions between silicon and the electrolyte in a battery [19,20].

**Figure 6:**
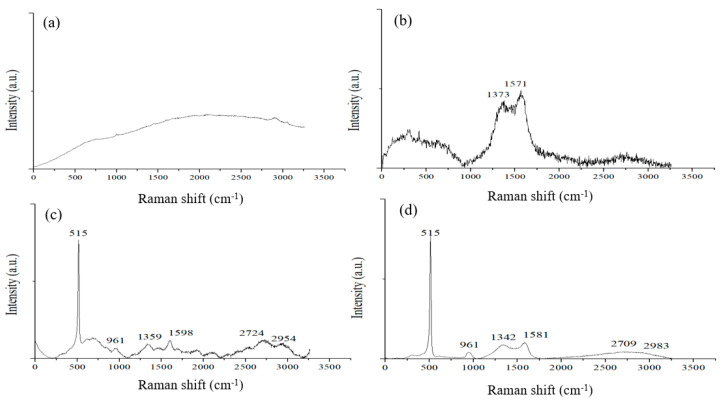
Raman spectra excited by 532 nm laser measured from (**a**) CMC/SBR film; (**b**) Pyrolytic CMC/SBR film; (**c**) Electrode made of silicon flakes and CMC/SBR binder of a weight ratio of 70%:30%; and (**d**) the electrode in (**c**) after pyrolysis.

Raman spectroscopy was applied to analyze the binder before and after pyrolysis. Binder made by a ratio of CMC/SBR = 1:1 in weight was prepared to coat on a copper foil without silicon powder. After the binders were dried, Raman spectroscopy was applied to differentiate the carbon contents of the binders before and after pyrolysis. Figure 6a shows a Raman spectrum of a CMC/SBR film before pyrolysis. The as-coated binder mixture is of amorphous nature and does not exhibit distinct characteristic peaks. After pyrolysis, the polymer film changed color to black. Broad D and G bands appeared in the Raman spectrum shown in Figure 6b. This is consistent with the structured carbon displayed by TEM images [21].

Figure 6c,d shows Raman spectra of an electrode made of silicon flake and the binder mixture, of which the Raman spectra are shown in Figure 6a,b before and after pyrolysis, respectively. The characteristic peaks of silicon crystals are clearly shown at the wavenumbers of 515 and 961 cm^−1^. Before pyrolysis, the electrode exhibits several characteristic peaks of carbon phases in the range of 550–900 and 1200–3000 cm^−1^ as shown in Figure 6c. After pyrolysis, most of the peaks disappeared, leaving broad D and G bands as shown in Figure 6d. The characteristic D and G bands prove that disordered graphitic structures were produced by the pyrolysis.

**Figure 7:**
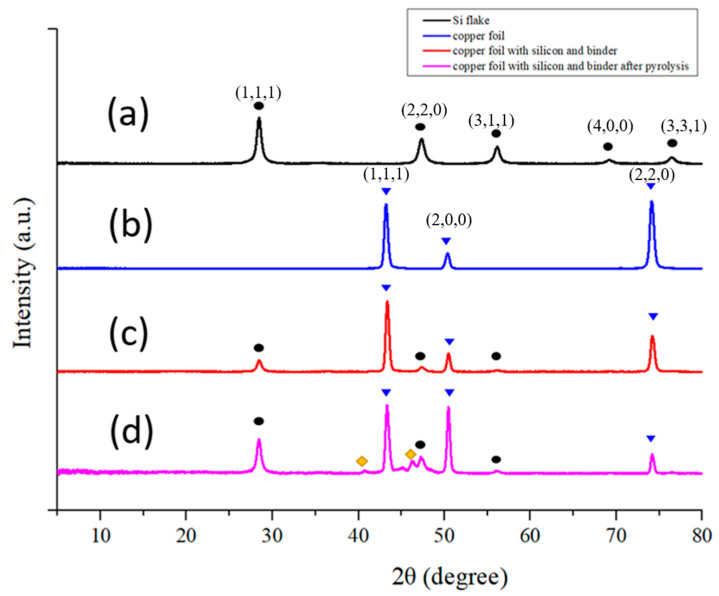
XRD Patterns of (**a**) Si flake, (**b**) Copper foil, (**c**) Si based anode without conductivity enhancement additive, and (**d**) Pyrolytic Si based anode without conductivity enhancement additive. Peaks marked by the same symbols belong to the same crystal facets. Peaks which are marked in yellow are unidentified.

Figure 7 shows XRD diffraction patterns of (a) a Si flake, (b) a copper foil, (c) a Si-based anode without conductivity enhancement additive, and (d) a Si-based anode without conductivity enhancement additive after pyrolysis, respectively. Figure 7a shows 2θ = 23°, 47°, 56, 69°, and 76° corresponding to interlayer space and planes of crystalline silicon. Figure 7b shows 2θ = 43°, 51°, and 74° corresponding to the d-space and planes of crystalline copper. Both CMC and SBR exhibit a broad peak near 2θ = 21°, indicating amorphous nature of both binders [22].

After pyrolysis, most of the binder was lost, leaving only 14 wt% of its original weight. In other words, the weight ratio of silicon powder and carbon material on the electrode became 94.3%:5.7%. Before pyrolysis, the conductivity enhancement additive is in an amorphous nature and there is no specific signal on XRD diffraction pattern. After pyrolysis, most of the Raman scattering signal from conductivity enhancement additive disappeared. The silicon contents and signal intensity of silicon powder are stronger as shown in Figure 7d.

**Figure 8:**
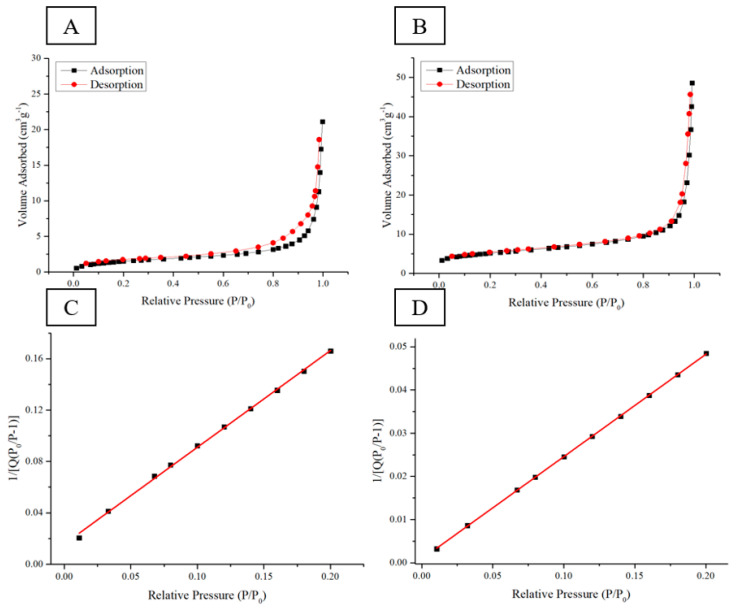
(**A**,**C**) Nitrogen adsorption–desorption isotherms of electrode with Si–CMC/SBR = 70%:30% in wt—before pyrolysis. (**B**,**D**) Nitrogen adsorption–desorption isotherms of electrode with Si–CMC/SBR = 70%:30% in wt—after pyrolysis.

Two circular ingot samples with a weight of 0.5 g and the weight ratio of Si–CMC–SBR being 70%:15%:15% were prepared for measuring the effective surface area before and after pyrolysis. Figure 8A,C shows nitrogen adsorption–desorption isotherms of electrode with Si–CMC/SBR = 70%:30% in wt before pyrolysis. Figure 8B,D shows nitrogen adsorption–desorption isotherms of electrode with Si–CMC/SBR = 70%:30% in wt after pyrolysis. The sample after having been subjected to pyrolysis lost weight with the remaining weight being 0.36 g. BET isotherm adsorption method was applied to examine the specific surface area. The pyrolysis process increased the specific surface area from 5.66 m^2^/g to 18.3 m^2^/g. It showed that the thermogravimetric loss of the binder does result in a porous electrode. The pores provide buffer space for silicon volume expansion during the formation of silicon–lithium compounds.

EIS measurement provides a variety of effective, time-varying information, including lithium-ion migration, carrier exchange, phase transfer, solid material transfer phenomenon, and the performance of capacitance effect. It is the most effective tool for observing the changes in the interface between the electrolyte and the electrode.

**Figure 9:**
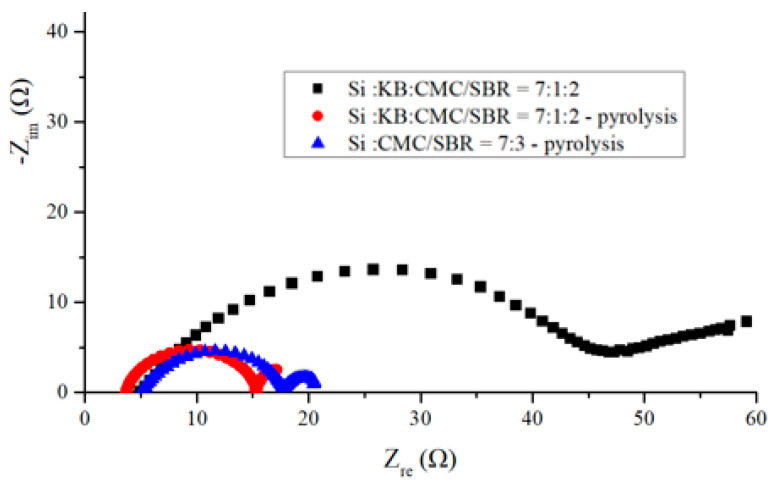
Electrochemical impedance spectra of anode before discharge–charge cycling experiments measured from three sets of anodes made of (black square) electrode with silicon flake: KB–CMC/SBR = 70%:10%:20% in weight ratio; (red dots) pyrolytic electrode with silicon flakes: KB–CMC/SBR = 70%:10%:20% in weight ratio; (blue triangle) pyrolytic electrode with silicon flakes–CMC/SBR = 70%:30% in weight ratio without conductivity enhancement additive.

Figure 9 shows the electrochemical impedance spectra of three anodes before cycling. The black squared curve was measured from an anode made according to the weight ratio of Si–KB–CMC/SBR = 70%:10%:20%. The spectrum representing the same anode after pyrolysis is presented by the red dotted curve. The blue triangle curve represents the spectrum for an anode made according to the weight ratio of Si–CMC/SBR = 70%:30% without KB additive and after pyrolysis.

The left side of each curve was measured at higher frequency than the right side. The bottom left point on the real number axis represents the contact resistance. The radius of the EIS semicircle stands for the carrier transfer resistance. The above impedance reflects the rate of insertion of lithium ions through the electric double-layer interface. The slope of the oblique line in the low-frequency region on the right side of the graph is related to the diffusion rate of lithium ions. The higher the slope of the oblique line is, the faster the diffusion rate of lithium ions in the sample [23].

For contact resistance, pyrolytic anode with conductivity enhancement additive exhibited the lowest value, and the pyrolytic anode without conductivity enhancement additive exhibits the largest value. This is consistent with the contents of conductive carbon in the anode. The contact impedances of three kinds of anodes are all below 5 ohms. On the other hand, pyrolytic binder provides a conductive network, which decreased the charge transfer resistance effectively.

**Figure 10:**
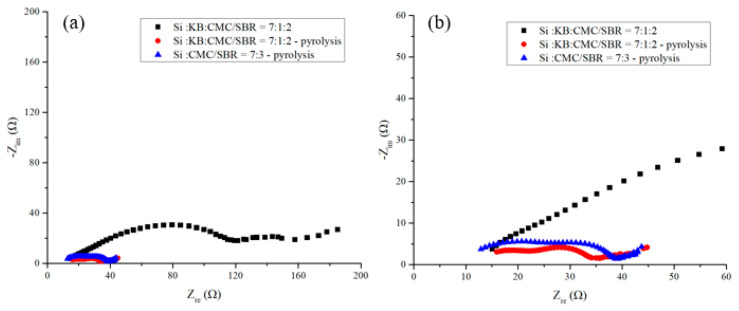
(**a**,**b**) Electrochemical impedance spectra measured from as fabricated anode and after 200 cycles of discharge/charge at 1000 mA/g for anode made of (black square) electrode with silicon flakes–KB–CMC/SBR = 70%:10%:20% in weight; (red dots) pyrolytic electrode with silicon flakes–KB–CMC/SBR = 70%:10%:20% in weight; (blue triangle) pyrolytic electrode with silicon flakes–CMC/SBR = 70%:30% in weight. (**b**) is an enlarged figure of (**a**).

Figure 10 shows the EIS spectra of anodes after 200 cycles of charge–discharge. The carrier transfer resistance of the silicon-based anode without pyrolysis increases to about 3 times of that before cycling. The sum of the radius of the first and second semicircles of the anode doubled. The slopes of the oblique straight lines in the low frequency area of the three curves declined. It indicates that the lithium-ion diffusion capability of the three anode decreased after 200 cycles of charging and discharging. Among them, the pyrolytic sample with 10 wt% KB exhibits the highest slope of the diagonal line and survives the longest cycling life. Pyrolytic anodes with conductivity enhancement additive exhibit improved rate of carrier transfer and reduced interface resistance effectively. The carrier transfer impedance in intermediate frequency of pyrolytic electrode with KB is slightly lower than that of the electrode without conductive enhancement agent. This is attributed to the promotion of the conductivity enhancement additives on the carrier transport. The cycling life of the silicon-based anode improves, too.

**Figure 11:**
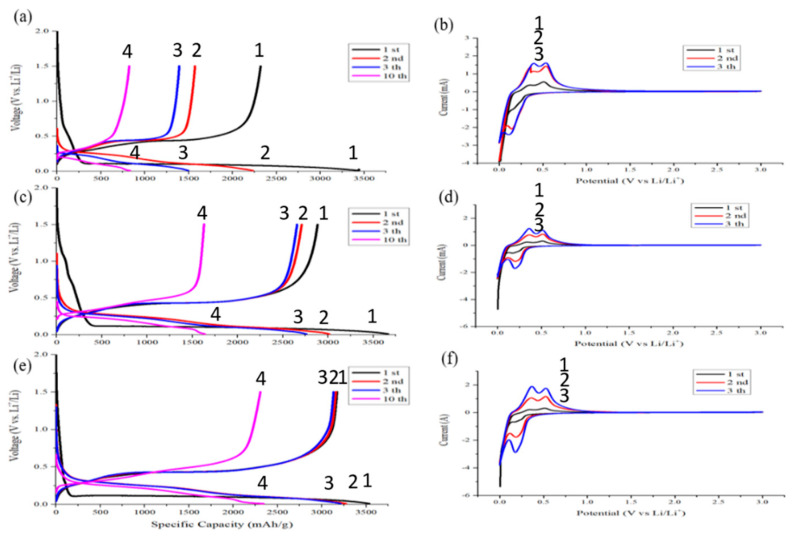
Cyclic Voltammetry measurements of anodes made of (**a**,**b**) silicon flakes: KB–CMC/SBR = 70%:10%:20% in weight ratio, (**c**,**d**) silicon flakes–KB–CMC/SBR = 70%:10%:20% in weight ratio after pyrolysis, and (**e**,**f**) silicon flakes–CMC/SBR = 70%:30% in weight ratio after pyrolysis. The scan rate is 0.1 mV/s. The discharge–charge rate for (**b**,**d**,**f**) is 200 mA/g for first three cycles and 1000 mA/g for the remaining cycles.

The initial coulombic efficiency of the pristine silicon anode is only 67.4%, and the capacity decay in the second and third cycles is fast. The 3rd charging capacity is only 60.8% of the initial charging. After the 10th charging and discharging, the capacity of this sample has only 780 mAh/g left. After pyrolysis, the same sample exhibits an initial coulombic efficiency that increased by 10.6%. The rate of capacity decline also slows down. The 3rd charging capacity is 96.4% of that of the first charging. After 10 times of cycling, this sample still retained a capacity of 1750 mAh/g. Moreover, the initial coulombic efficiency of pyrolytic anode even without conductive agent is as high as 89.6%. The 3rd charging capacity of the sample is almost the same as the first one as shown in Figure 11e. The pyrolysis process not only increases the first cycle coulombic efficiency but also extends the cycling life of the silicon-based anode.

Figure 11a,c,e shows that the area of the 1st corresponding capacitance before the potential drops to 0.01 V is proportional to the nanocarbon contents, which are present in the anode. Less nano-carbon is desirable to avoid the side effects between nano-carbon and electrolyte in the first stage of the cycling. The less conductive carbon in the anode, the higher the initial coulombic efficiency will be. To optimize the ICE, either the nano-carbon contents need to decrease, or a better choice of nano-carbon phase is desirable.

Figure 11b,d,f shows discharge–charge CV curves. The discharge begins with the lithiation of the crystalline silicon near zero volt. The lithiation forms lithium–silicon alloys. When alloys dissociate to release lithium, silicon does not recrystallize but instead becomes amorphous silicon. The delithiation curves exhibit two peaks near 0.4 V and 0.6 V. These peaks are attributed to a two-step delithiation process, i.e., the anode first delithiates to become an intermediate lithium silicon alloy [24].

The cyclic voltammetry patterns of the pyrolytic samples are similar. The scanning patterns of the two and three circles are basically unchanged. This indicates that the reversibility of the pyrolytic anode is good. The pyrolysis process does improve the electrical performance of an anode.

**Figure 12:**
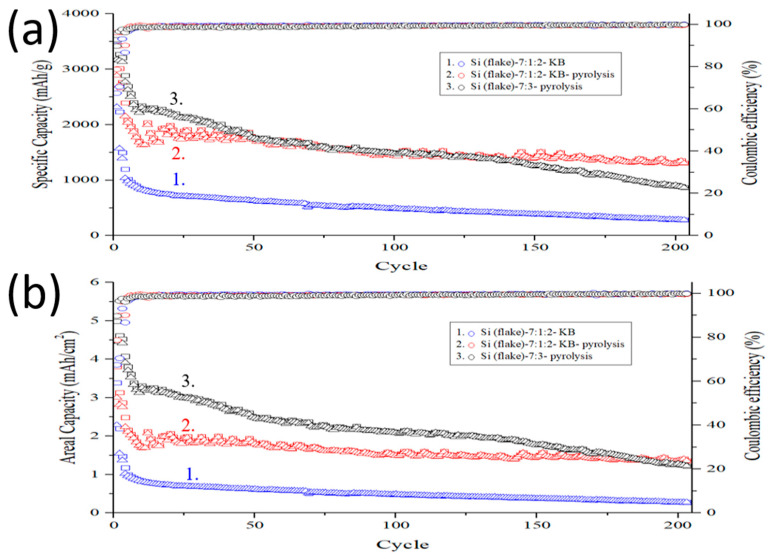
(**a**) Specific capacity and (**b**) areal capacity cycling performance of anodes made of (black, 3) silicon flakes–CMC/SBR = 70%:30% in weight ratio after pyrolysis; (red, 2) silicon flakes–KB–CMC/SBR = 70%:10%:20% in weight ratio after pyrolysis; (blue, 1) silicon flakes–KB–CMC/SBR = 70%:10%:20% in weight ratio without pyrolysis.

Figure 12 shows cycling performance of an anode made of Si flake–KB–CMC/SBR = 70%:10%:20% in weight ratio, a pyrolytic anode made of Si flake–KB–CMC/SBR = 70%:10%:20% in weight ratio, and a pyrolytic anode made of Si flake–CMC/SBR = 70%:30% in weight ratio. The mass loading of these anodes is in the range of 1.2–1.5 mg/cm^2^. The charge–discharge cycling current is 200 mA/g for the first three cycles and 1000 mA/g for the subsequent cycles. Graphitic carbon coating on silicon flake reduces direct contact of silicon with the electrolyte and their undesirable irreversible reactions. It results in much improved initial coulombic efficiency.

When conductivity enhancement additive (KB) was not added, there was only 5.7 wt% of carbon that was formed by conversion from the binder by the pyrolysis process. The low nanocarbon contents further reduce undesirable reactions with electrolyte catalyzed by nano carbon. The achieved initial coulombic efficiency was as high as approximately 90%.

The anode made of silicon flake, binder, and conductivity enhancement additives but without pyrolysis process lacks graphitic carbon coatings on silicon flakes and a porous structure of pyrolyzed binder. The conductivity of this anode is the lowest among three anodes. Silicon volume expansion leads to high internal stresses, which tend to cause cracks in the anode and losses of electrical connectivity between cracked anode pieces. As a result, such an anode exhibits poor electrochemical performance with the capacity rapidly declining to 500 mAh/g after only about 10 cycles of charging and discharging. After 200 charging and discharging cycles, the capacity of silicon anode decreases to 250 mAh/g.

In contrast, pyrolytic anodes exhibit slow decay in the capacity by cycling. This is attributed to the graphitic carbon coatings on silicon flake and the porous conductive carbon structure formed by the binder pyrolysis process. The electrochemical performance of two pyrolytic anodes studied are different. In the stage of the 1st–10th cycles, the pyrolytic anode with Si flake–KB–CMC/SBR = 70%:10%:20% in weight ratio decayed faster than the pyrolytic anode with Si flake–CMC/SBR = 70%:30% in weight ratio. The KB conductivity enhancement additive acts as an undesirable catalyst for reactivity of the electrolyte and results in reduced ICE and faster decay in the capacity cycling. In this respect, an anode without KB is desirable. A different conductivity enhancement additive than KB or a less amount of KB should have been applied.

However, in the later stage of the capacity cycling, the capacity of the pyrolytic anode with Si flake–KB–CMC/SBR = 70%:10%:20% in weight ratio remained basically constant with only a smaller decrease than the anode without KB additive. After 200 cycles, the anode still maintained a high capacity of nearly 1200 mAh/g, and a high areal capacity of 1.4 mAh/cm^2^. As a comparison, the capacity of the pyrolytic anode made of Si flake–CMC/SBR = 70%:30% in weight ratio without KB additive declines rapidly in the initial 50 cycles. Without conductivity enhancement additive, KB, although the anode performs well, the conductivity of the anode is too low to handle the high cycling current of 1 A/g. In the 50th–150th cycles, the capacity of this sample remained stable with only slightly decreased to 1.75 mAh/cm^2^. After 150 cycles, the capacity of the sample gradually decreased. After 200 cycles, the sample retains a capacity of 900 mAh/g (1.3 mAh/cm^2^). Without the conductivity enhancement additive, KB, the internal resistance gradually increases with repetitive charging and discharging causing the capacity to decline.

**Figure 13:**
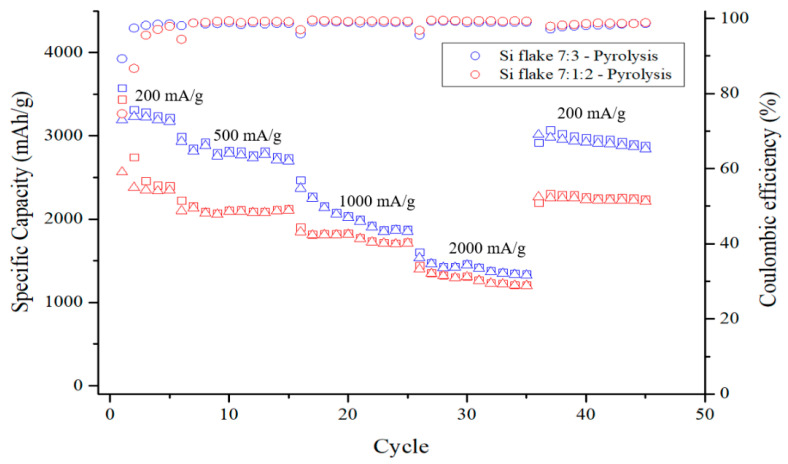
Cycling performance of an anode made of pyrolytic silicon anodes with (1) silicon flake–binder = 70%:30% in weight ratio and (2) silicon flake–KB–binder = 70%:10%:20% in weight ratio at discharge–charge rates varied from 0.2A/g (0.05 C) to 2A/g (0.48 C). The discharge–charge rate is based on the weight of silicon flakes.

Figure 13 shows the capacity cycling and the coulombic efficiencies of two kinds of anodes after having been subjected to pyrolysis at different charging–discharging rates. The test current of the measurement is 200 mA/g for the first 5 cycles, followed by 500 mA/g, 1000 mA/g, and 2000 mA/g for 10 cycles each. The specific capacity of the pyrolytic anodes is higher than 2200 mAh/g with the anode without conductivity enhancement additive, KB, being as high as 3000 mAh/g. The difference is due to the different ICE of these two anodes. The ICE of anode containing KB is only 75%, while the anode without KB exhibits an ICE is as high as 89%. The conductivity enhancement additive promotes the conductivity of the anode but also consumes significant lithium during the first discharge–charge cycles. An optimized small amount of conductivity enhancement additive is desirable. When the discharge–charge current is increased to 1000 mA/g, the capacity of the anode with conductivity enhancement additive, KB, does not decline significantly, while the anode without KB exhibits a faster decline in capacity due to low internal conductivity of the anode. When the current is restored to 200 mAh/g, the capacity increased to 2300 mAh/g and 3000 mAh/g, for anode without and with KB, respectively. The capacity decreased by less than 10% after the cycling at different current rate up to 2 A/g. These results show that pyrolytic silicon-based anodes exhibit excellent electrochemical performance and capacity retention.

**Figure 14:**
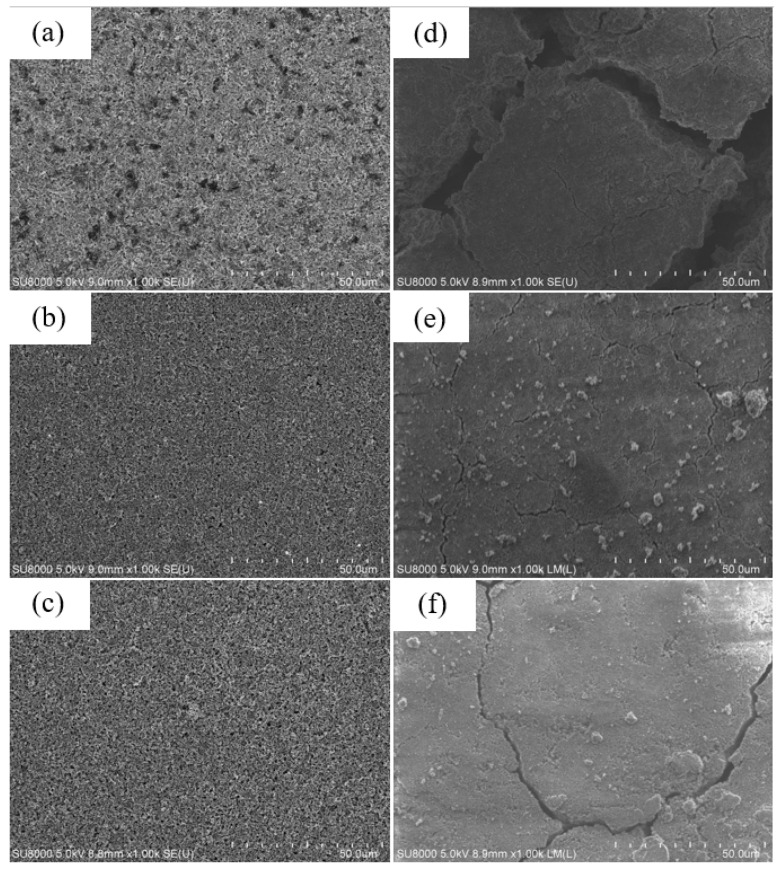
SEM images of (**a**,**d**) anode made of silicon flakes–KB–CMC/SBR = 70%:10%:20% in weight ratio, (**b**,**e**) pyrolytic anode made of silicon flakes–KB–CMC/SBR = 70%:10%:20% in weight ratio, (**c**,**f**) pyrolytic anode made of silicon flakes–CMC/SBR = 70%:30% in weight ratio (**a**–**c**) before, and (**d**–**f**) after 200 discharge–charge cycles.

Figure 14 shows SEM images of the surface of (a) as-fabricated anode made of Si–KB–CMC/SBR = 70%:10%:20% in weight ratio, (b) pyrolytic anode made of Si–KB–CMC/SBR = 70%:10%:20% in weight ratio, and (c) pyrolytic anode made of Si–CMC/SBR = 70%:30% in weight ratio. The anodes were cleaned by DEC solvent, in order to achieve the consistency of the image. The surfaces of the as-fabricated anodes were homogeneous without cracks.

For an anode without pyrolysis, after repetitive discharge–charge cycling, volume changes of silicon flake might cause cracks appearing on the surface of the anode, as shown in Figure 14d. In the contrast, the pyrolyzed anodes contain carbonized binder, which was not only beneficial to the anode conductivity, but also coated on the surface of the silicon flake with graphitic carbon as a barrier against undesirable reactions between silicon surface and the electrolyte, reduced side reactions, and provided room in the porous carbon structure as a buffer for the volume changes of silicon flakes. As a result, the surface cracks of pyrolytic anodes shown in Figure 14e,f after 200 cycles are relatively small compared to those of the anode without pyrolysis as shown in Figure 14d. Small cracks are more likely shallow without completely losing electrical connectivity. Large cracks cause two sides of anode to lose electrical contacts.

Figure 14f shows the surface morphology of a pyrolytic electrode made of Si flake–CMC/SBR = 70%:30% in weight ratio without conductivity enhancement additive, KB, but instead, only containing 5.7% of conductive carbon by weight that was formed by the pyrolysis of the binder. Although a smaller amount of conductive carbon reduces the reactions catalyzed by nano-carbon and effectively improve the ICE, the lithium-ion diffusion rate is gradually reduced during the capacity cycling. This is not desirable for long-term cycling. By adding a small but appropriate amount of conductivity enhancement additive, desirable diffusion rate of lithium ions during the cycling is preserved to increase the cycling life of the anode while keeping the ICE near 90%. Figure 14e shows the surface morphology of an example of such an optimized anode.

**Figure 15:**
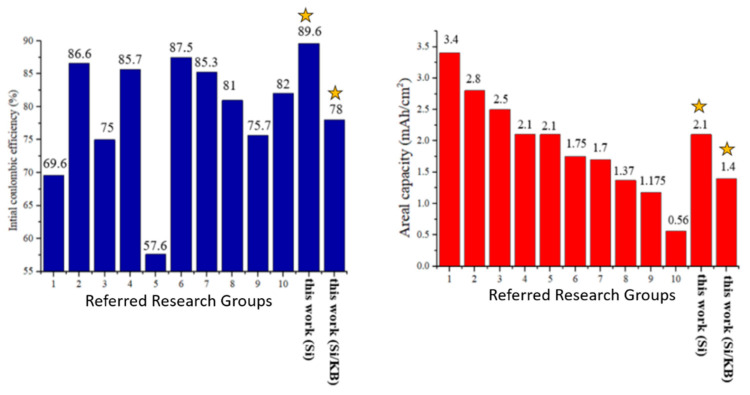
Comparison of initial coulombic efficiency (blue bars) and areal capacity (red bars) of selected high-performance anodes reported in the literature. Referred research group #1 is [25] and group #2 is [26] and so on. Bars for this work are marked by yellow stars.

Figure 15 compares this work with selected high-performance silicon-based anodes which have been published in the literature in recent years [25,26,27,28,29,30,31,32,33,34]. The initial coulomb efficiency and the 100th areal capacity are compared. Research groups #1 [25], #2 [26], and #4 [28] reported higher areal capacity using silicon powder of smaller than 100 nm in size to reduce the stress issues induced by silicon volume expansion. Group #3 [27] used magnesium powder to reduce the coating layer of silicon dioxide, followed by reducing silicon dioxide to form silicon as the active material. Group #5 [29] used commercial SiOx powder as the active material for the anode. High-areal capacity silicon-based anode was achieved by some groups partially by means of choosing appropriate silicon materials and sizes to overcome the problems of silicon volume expansion and pulverization during charging and discharging cycling. However, the costs of the synthesis and purification processes for producing nano-sized silicon powder and SiOx and the magnesium reduction process are high.

Silicon flake (800 nm × 800 nm × 100 nm) recycled from silicon wafer manufacturing waste was used as the active material in this work. By pyrolyzing the binder, excellent initial coulombic efficiency of silicon-based anode of about 90% was achieved. This is higher than or as good as data reported in recent publications. We attribute this excellent performance to firstly, micron-sized flat silicon flake exhibits smaller effective surface area than silicon nanoparticles of the same total volume. Secondly, the graphitic coating on silicon by pyrolyzing the binder protects silicon surface from undesirable reactions with the electrolyte to form irreversible silicon-lithium compounds. The silicon anode prepared in this work retains a high areal capacity of 2.1 mAh/cm^2^ after 100 discharge–charge cycles. It maintained an areal capacity of more than 1.4 mAh/cm^2^ after 200 cycles.

Mass loading of silicon-based anodes are typically in the range of 1–2 mg/cm^2^ [25,26,27,28,29,30,31,32,33,34]. An anode with a higher loading suffers from long lithium ions diffusion time. Group #3 [27] made the anode with a mass loading of more than 4 mg/cm^2^, but the electrochemical performance of the two samples with 4.0 mg/cm^2^ and 4.9 mg/cm^2^ is not as good as the sample with only 3 mg/cm^2^. In addition, group #9 [33] also made the electrode with a loading of more than 10 mg/cm^2^. As a result, the capacity of this anode dropped significantly near the beginning stage of the discharge–charge cycling. In this work, mass loading in the range of 1.2–1.5 mg/cm^2^ was chosen. KB was used as the conductivity enhancement additive. There is no pre-fabrication coating of carbon or other protective layer on silicon. For our anodes made without pyrolysis, the ICE was only 67.4%, and the areal capacity decreased to only 0.35 mAh/cm^2^ after 100 cycles.

After pyrolyzing the anode, The ICE increased to 78.0%, and after the 100th cycle, the areal capacity was 1.4 mAh/cm^2^. There was little decline in capacity afterwards. The excellent electrochemical performance may be attributed to the graphitic carbon coatings on the surface of the silicon flake. The coatings serve as protective layers to avoid side reactions of silicon, especially at the initial stage of the cycling, with the electrolyte. At the same time, the binder transformed into a porous carbon structure, providing additional buffer room for volume expansion of the silicon flake, improving the cycling lifetime of the anode.

Higher ICE than 78% is desirable. Reactions of silicon with electrolyte is not the only major cause to the consumption of lithium in the first cycle. To investigate the roles played by the conductivity enhancement additive, KB, on the ICE, pyrolytic anode without KB was fabricated. Without KB, the ICE increased from 78% to 89.6%, and after the 100th cycle the areal capacity was 2.1 mAh/cm^2^. We attribute the significant increase in ICE to the absence of KB as a catalyst for undesirable reactions with the electrolyte during the initial discharging and charging cycle. Therefore, some active carbon contents in the anode have adverse effects on ICE. The pyrolytic anode made of Si flake–KB–CMC/SBR = 70%:10%:20% in weight ratio contains 15.5 wt% nano carbon and exhibits ICE of 78.0%. As a comparison, pyrolytic anode made of Si flake–CMC/SBR = 70%:30% in weight ratio contains only 5.7 wt% nanocarbon and exhibits a higher ICE of 89.6%.

However, from Figure 9 and Figure 10 it shows that the lithium-ion diffusion rate of the anode without KB additive is poorer than that with KB and the capacity retention after long cycling time is lower than that of the pyrolytic anode with KB. Although the porous carbon structure provides sufficient conductivity and physical integrity for the anode to perform better than the anode with KB but without pyrolysis, the conductivity was not adequate for the high demanding support of the charge–discharge cycling. When the anode internal impedance increases with cycling time, cracks appeared and the capacity decay rate increased in the cycling. KB was chosen as the conductivity enhancement additive for this study because of its high electrical conductivity. An optimized amount of KB additive or by choosing a different inert conductivity enhancement additive for the pyrolytic anode is believed to be able to achieve a long-cycling-life anode with a high ICE on the order of 90%. Indeed, when the KB is replaced by Super P conductivity enhancement additive, the pyrolytic anode made of Si flake–Super P–CMC/SBR = 70%:10%:20% in weight ratio exhibited an excellent ICE of 89.92%. Research is being undertaken to study the effects of different kinds and amounts of conductivity enhancement additives on ICE. The detailed results will be published in the near future.

## 4. Conclusions

Silicon flakes of about 100 nm × 800 nm × 800 nm in size were recycled from wastes of silicon wafer manufacturing processes for the fabrication of silicon-based anode for lithium-ion battery. Pyrolysis of the anode resulted in conversion of the binder in contact with silicon surface to graphitic carbon coatings with the binder forming a porous carbon structure to provide physical integrity and internal conductivity of the anode. The anode made of silicon flake–KB–(CMC/SBR) = 70%:10%:20% in weight ratio and that made of silicon flake–(CMC/SBR) = 70%:30% in weight ratio both exhibited excellent cycling performance in comparison with an anode without being subjected to the pyrolysis. The pyrolyzed anode without conductivity enhancement additive, KB, exhibited the highest ICE about 90% and an areal capacity of 2.1 mAh/cm^2^ after the 100th cycle. However, the areal capacity decreased to 1.3 mAh/cm^2^ after 200 cycles due to the gradual increase of internal resistance of the anode. A compromise between high ICE of 90% and high-capacity retention rate after a long cycling life can be achieved by the optimized addition of conductivity enhancement additive, KB, or the choice of conductivity enhancement additive that does not cause undesirable reactions of the electrolyte during the initial discharge–charge cycling. In both cases with and without the addition of conductivity enhancement additive, KB, pyrolysis of the anode significantly improves the capacity retention rate and increases the ICE.

An environmentally friendly and simple process for the fabrication of high-performance silicon-based anode has been demonstrated. The pyrolysis process provides one step coating of inexpensive and abundant silicon flake with graphitic carbon and formation of a porous carbon structure for supporting the physical integrity and at the same time providing buffer room for silicon volume changes during charging and discharge. Pyrolysis is a facile and promising one-step process for the fabrication of silicon-based anode with high ICE and long cycling life when the choice of an inert conductivity enhancement additive is optimized.

## Data Availability

Data can be found in our Lab notes.

## References

[1] Jung H., Park M., Yoon Y., Kim G., Joo S. (2003). Amorphous silicon anode for lithium-ion rechargeable batteries. J. Power Sources.

[2] Amine K., Kanno R., Tzeng Y. (2014). Rechargeable lithium batteries and beyond: Progress, challenges, and future directions. MRS Bull..

[3] Zhang W., Truong H.C., Brian W.S. (2019). The Impact of Initial SEI Formation Conditions on Strain-Induced Capacity Losses in Silicon Electrodes. Adv. Energy Mater..

[4] Hatchard T.D., Dahn J.R. (2004). In Situ XRD and Electrochemical Study of the Reactions of Lithium with Amorphous Silicon. J. Electrochem. Soc..

[5] Obrovac M.N., Christensen L. (2004). Structural Changes in Silicon Anodes during Lithium Insertion/Extraction. Electrochem. Solid-State Lett..

[6] Cheng Y.-W., Lin C.-K., Chu Y.-C., Abouimrane A., Chen Z., Ren Y., Liu C.-P., Tzeng Y., Auciello O. (2014). Electrically Conductive Ultrananocrystalline Diamond-Coated Natural Graphite-Copper Anode for New Long Life Lithium-Ion Battery. Adv. Mater..

[7] Tzeng Y., Huang W.-C., Jhan C.-Y., Wu Y.-H. (2021). Effects of In Situ Graphitic Nanocarbon Coatings on Cycling Performance of Silicon-Flake-Based Anode of Lithium Ion Battery. Coatings.

[8] Tzeng Y., He J.-L., Jhan C.-Y., Wu Y.-H. (2021). Effects of SiC and Resorcinol–Formaldehyde (RF) Carbon Coatings on Silicon-Flake-Based Anode of Lithium Ion Battery. Nanomaterials.

[9] Tzeng Y., Chen R., He J.-L. (2020). Silicon-Based Anode of Lithium Ion Battery Made of Nano Silicon Flakes Partially Encapsulated by Silicon Dioxide. Nanomaterials.

[10] Yu C., Chen X., Xiao Z., Lei C., Zhang C., Lin X., Shen B., Zhang R., Wei F. (2019). Silicon carbide as a protective layer to stabilize Si-based anodes by inhibiting chemical reactions. Nano Lett..

[11] Mi H., Yang X., Li Y., Zhang P., Sun L. (2018). A self-sacrifice template strategy to fabricate yolk-shell structured silicon@void@carbon composites for high-performance lithium-ion batteries. Chem. Eng. J..

[12] Xie J., Tong L., Su L., Xu Y., Wang L., Wang Y. (2021). Core-shell yolk-shell Si@C@Void@C nanohybrids as advanced lithium ion battery anodes with good electronic conductivity and corrosion resistance. J. Power Sources.

[13] Liu X.H., Zhong L., Huang S., Mao S.X., Zhu T., Huang J.Y. (2021). Size-dependent fracture of silicon nanoparticles during lithiation. ACS Nano.

[14] Ryu I., Choi J.W., Cui Y., Nix W.D. (2011). Size-dependent fracture of Si nanowire battery anodes. J. Mech. Phys. Solids.

[15] Xu Y., Yin G., Ma Y., Zuo P., Cheng X. (2010). Simple annealing process for performance improvement of silicon anode based on polyvinylidene fluoride binder. J. Power Sources.

[16] Jiao X., Yin J., Xu X., Wang J., Liu Y., Xiong S., Zhang Q., Song J. (2021). Highly Energy-Dissipative, Fast Self-Healing Binder for Stable Si Anode in Lithium-Ion Batteries. Adv. Funct. Mater..

[17] Kim H.S., Chung K.Y., Cho B.W. (2009). Electrochemical properties of carbon-coated Si/B composite anode for lithium ion batteries. J. Power Sources.

[18] Liu J., Kopold P., van Aken P.A., Maier J., Yu Y. (2015). Energy storage materials from nature through nanotechnology: A sustainable route from reed plants to a silicon anode for lithium-ion batteries. Angew. Chem..

[19] Fang G., Deng X., Zou J., Zeng X. (2019). Amorphous/ordered dual carbon coated silicon nanoparticles as anode to enhance cycle performance in lithium ion batteries. Electrochim. Acta.

[20] Zhou S., Fang C., Song X., Liu G. (2020). The influence of compact and ordered carbon coating on solid-state behaviors of silicon during electrochemical processes. Carbon Energy.

[21] Hodkiewicz J., Scientific T.F. (2010). Characterizing Carbon Materials with Raman Spectroscopy. Sci. Appl. Note.

[22] Morsia M.A., Abdelaziz M., Oraby A.H., Mokhles I. (2019). Structural, optical, thermal, and dielectric properties of polyethylene oxide/ carboxymethyl cellulose blend filled with barium titanate. J. Phys. Chem. Solids.

[23] Ouyang P., Jin C., Xu G., Yang X., Kong K., Liu B., Zhou L. (2021). Lithium ion batteries with enhanced electrochemical performance by using carbon-coated SiOx/Ag composites as anode material. Ceram. Int..

[24] Liu J., Kopold P., van Aken P.A., Maier J., Yu Y. (2015). Uniform yolk–shell Sn_4_P_3_@C nanospheres as high-capacity and cycle-stable anode materials for sodium-ion batteries. Energy Environ. Sci..

[25] Han M., Lin Z., Ji X., Mu Y., Li J., Yu J. (2020). Growth of flexible and porous surface layers of vertical graphene sheets for accommodating huge volume change of silicon in lithium-ion battery anodes. Mater. Today Energy.

[26] Zhang X., Wang D., Qiu X., Ma Y., Kong D., Müllen K., Zhi L. (2020). Stable high-capacity and high-rate silicon-based lithium battery anodes upon two-dimensional covalent encapsulation. Nat. Commun..

[27] Jia H., Li X., Song J., Zhang X., Luo L., He Y., Zhang J.G. (2020). Hierarchical porous silicon structures with extraordinary mechanical strength as high-performance lithium-ion battery anodes. Nat. Commun..

[28] Liu H., Chen T., Xu Z., Liu Z., Yang J., Chen J. (2020). High-Safety and Long-Life Silicon-Based Lithium-Ion Batteries via a Multifunctional Binder. ACS Appl. Mater. Interfaces.

[29] Wu S., Yang Y., Liu C., Liu T., Zhang Y., Zhang B., Lin Z. (2020). In-Situ Polymerized Binder: A Three-in-One Design Strategy for All-Integrated SiO x Anode with High Mass Loading in Lithium Ion Batteries. ACS Energy Lett..

[30] Wang J., Liao L., Lee H.R., Shi F., Huang W., Zhao J., Cui Y. (2019). Surface-engineered mesoporous silicon microparticles as high-Coulombic-efficiency anodes for lithium-ion batteries. Nano Energy.

[31] Li B., Zhao W., Yang Z., Zhang C., Dang F., Liu Y., Chen X. (2020). A carbon-doped anatase TiO2-Based flexible silicon anode with high-performance and stability for flexible lithium-ion battery. J. Power Sources.

[32] Tao Y., Zeng G., Xiao C., Liu Y., Qian Y., Feng J. (2019). Porosity controlled synthesis of nanoporous silicon by chemical dealloying as anode for high energy lithium-ion batteries. J. Colloid Interface Sci..

[33] Gao S., Sun F., Brady A., Pan Y., Erwin A., Yang D., Cao P.F. (2020). Ultra-efficient polymer binder for silicon anode in high-capacity lithium-ion batteries. Nano Energy.

[34] Mishra K., George K., Zhou X.D. (2018). Submicron silicon anode stabilized by single-step carbon and germanium coatings for high capacity lithium-ion batteries. Carbon.

